# Isolation and characterization of a high iturin yielding *Bacillus velezensis* UV mutant with improved antifungal activity

**DOI:** 10.1371/journal.pone.0234177

**Published:** 2020-12-03

**Authors:** Young Tae Kim, Sung Eun Kim, Won Jung Lee, Zhao Fumei, Min Sub Cho, Jae Sun Moon, Hyun-Woo Oh, Ho-Yong Park, Sung Uk Kim

**Affiliations:** 1 Industrial Bio-materials Research Center, Korea Research Institute of Bioscience and Biotechnology (KRIBB), Daejeon, Republic of Korea; 2 Molecular Biofarming Research Center, KRIBB, Daejeon, Republic of Korea; 3 Green Biotech Co., Paju, Republic of Korea; 4 Core Facility Management Center, KRIBB, Daejeon, Republic of Korea; Tallinn University of Technology, ESTONIA

## Abstract

To isolate *Bacillus velezensis* mutants with improved antifungal activity for use in the biological control of phytopathogenic fungi, wild-type *Bacillus velezensis* KRF-001 producing iturin, surfactin, and fengycin was irradiated by ultraviolet (UV) rays. The *in vitro* and *in vivo* antifungal activities of UV mutants and characterization of the cyclic lipopeptides produced by a selected mutant were examined. A mutant strain yielding high levels of iturin showed over 2-fold higher antifungal activity than the wild-type against *Fusarium oxysporum*. A potent suppressive effect of the mutant was also observed on spore germination of *Botrytis cinerea*, the causative agent of cucumber gray mold, at different butanol extract concentrations. Further analysis of the mutant by real-time PCR and high-performance liquid chromatography revealed increased expression of iturin and surfactin biosynthesis genes as well as enhanced production of iturin and surfactin metabolites. However, the amounts of fengycin obtained from the mutant strain BSM54 were significantly lesser than those of iturin and surfactin. Particularly, iturin A production by the mutant was 3.5-fold higher than that of the wild-type, suggesting that the higher antifungal activity of the mutant against *F*. *oxysporum* resulted from the increased expression of biosynthesis genes associated with iturin production. The commercial greenhouse experiment using soil naturally infested with *Sclerotinia sclerotiorum* (sclerotinia rot) and *F*. *oxysporum* (fusarium wilt) showed that the mutant strain reduced sclerotinia rot and fusarium wilt diseases (*P* = 0.05) more effectively than the wild-type and commercially available product Cillus® in Korea. These results suggest that the mutant with high iturin yield is a potential candidate for the development of a biological control agent in agriculture.

## Introduction

Continuous use of agrochemicals has contributed to an increase in crop production over the past few decades. However, their use is gradually being limited in a number of crops because of the harmful environmental effects and emergence of pesticide-resistant strains. Recently, environmentally friendly biological control agents as alternatives to agrochemicals have received attention because of their versatility in modes of action, efficacy, and potential to be used as complements to agrochemicals [1–5].

Members of the genus *Bacillus* are well-known producers of antimicrobials that can inhibit phytopathogen growth [1, 6, 7]. The advantages of *Bacillus* strains over other biological control microorganisms include their spore-forming abilities and resistance to extreme conditions in greenhouses or fields. Especially, *Bacillus subtilis* and *Bacillus amyloliquefaciens* strains have been extensively studied as biological control agents of plant pathogens [1, 8–10]. Some *Bacillus* strains, including *B*. *subtilis* and *B*. *amyloliquefaciens*, produce a variety of nonribosomally synthesized cyclic lipopeptides, including iturin, surfactin, and fengycin, which show potent antifungal activity against phytopathogenic fungi and can be used as biological control agents to protect plants [11–17], as well as fruits and vegetables from postharvest diseases [5, 18, 19]. Among the antifungal lipopeptides, iturins consist of heptapeptides linked to a ß-amino fatty acid chain that is 14–17 carbons in length and cause osmotic perturbations by forming ion-conducting pores that result in the leakage of K^+^ and other vital ions, precipitating cell death [20–22]. Iturins have shown strong antifungal activity against a wide variety of fungi, with limited antibacterial and no antiviral activities [21]. Surfactins are heptapeptides interlinked with a ß-hydroxy fatty acid chain and are powerful biosurfactants with exceptional emulsifying, foaming, antiviral, antimycoplasma, and hypocholesterolemic activities, but no apparent antifungal activities [14, 21, 23]. Surfactin exerts its cellular effects by altering membrane integrity [24] and shows synergism by forming mixed micelles with iturin [21, 23]. The fengycin family, including the closely related plipastatin, comprises decapeptides containing a ß–hydroxy fatty acid with a side-chain length of 16–19 carbon atoms [9, 15, 17, 25]. Fengycins alter the permeability of fungal cell membranes by interacting with sterol and phospholipid molecules in the membranes [26] and are specifically active against filamentous fungi [17, 27, 28].

In a previous study, wild-type *B*. *subtilis* subsp. *krictiensis* ATCC55079, which produces iturins A to F [29], showed potent antifungal activity against a variety of phytopathogenic fungi [30], suggesting the possibility of its application as a biological control agent. Recently, through high performance liquid chromatography (HPLC) and liquid chromatography/mass spectrometry (LC/MS) analyses, we found that this strain also produces a small amount of surfactins [31] and fengycins.

To improve the antifungal activity of antagonistic wild-type *B*. *velezensis* KRF-001 (formerly *B*. *subtilis* subsp. *krictiensis* ATCC55079) with increased iturin production, a wild-type *B*. *velezensis* strain was irradiated by ultraviolet (UV) rays and a mutant strain yielding high levels of iturin was selected on agar plates overlaid with soft agar containing *Fusarium oxysporum*. The antifungal activity of this mutant was compared with that of *B*. *subtilis* strains isolated from commercial products. In addition, the expression levels of iturin and surfactin biosynthesis genes in both wild-type and mutant strains were investigated by real-time PCR analyses, and the suppressive effects of these strains on *Botrytis cinerea* spore germination were examined. The difference in the secondary metabolites produced by wild-type *B*. *velezensis* and the mutant strain with high iturin yields was analyzed quantitatively with HPLC. The *in vivo* antifungal activities of the wild-type and mutant strains against phytopathogenic fungi were examined in a commercial greenhouse.

## Materials and methods

### Bacterial strains and culture conditions

Wild-type *Bacillus velezensis* KRF-001, the iturin, surfactin, and fengycin-producing strain used in this study, was isolated from soil as previously described [30, 31]. Wild-type UV mutants M1891 and UV4-II, previously isolated in our laboratory from wild-type *B*. *velezensis*, were stored at -70°C in 20% (vol/vol) glycerol. The *Bacillus* strains tested for antifungal activity were isolated from commercially available products purchased in Korea and United States, and their antifungal activities were compared with the UV mutant strains. These bacteria were also stored at -70°C in 20% glycerol. *Fusarium oxysporum* and *Botrytis cinerea*, which were used for antifungal activity and spore germination bioassays, respectively, were incubated at 25°C on potato dextrose agar (PDA). All bacteria were incubated at 30°C overnight in Luria-Bertani (LB) broth. The medium used to culture the *Bacillus* cyclic lipopeptide-producing strains was a complex medium containing sucrose (30.0 g/L), soytone (10.0 g/L), yeast extract (5.0 g/L), K_2_HPO_4_ (0.5 g/L), MgSO_4·_7H_2_O (2.0 g/L), MnCl_2_ (4.0 mg/L), CaCl_2_ (5.0 mg/L), and FeSO_4_·7H_2_O (25.0 mg/L) in distilled water, adjusted to pH 7.0.

### Molecular identification of wild-type *Bacillus* strain

Genomic DNA was extracted using the Nucleo^®^Spin Microbial DNA kit (Macherey-Nagel, Germany) according to the manufacturer’s standard protocol. PCR amplification of the 16S rRNA genes was performed using bacterial universal primers 27F (5′-AGA GTT TGA TCC TGG CTC AG-3′) and 1492R (5′-ACG GCT ACC TTG TTA CGA CTT-3′) [32]. The PCR band of approximately 1.4 kb in size was purified using Nucleo^®^Spin Gel and PCR Clean-up kit (Macherey-Nagel, Germany). The amplified 16S rRNAs were directly sequenced using a BigDye terminator cycle sequencing kit (Applied Biosystems, U.S.A.) according to the manufacturer’s instructions. BLAST searches revealed the closest matches with isolates on the NCBI GenBank database. DNA sequences were aligned using the Clustal X program and a phylogenetic analysis was performed using the Mega 7 program.

### UV irradiation

Wild-type UV mutant *B*. *velezensis* M1891 and UV4-II isolated from *B*. *velezensis* KRF-001 were grown at 30°C overnight in LB broth. Aliquots of the culture broths were inoculated into fresh LB broth and incubated for growth to an absorbance of 0.6 at a 550 nm wavelength. The culture broths of each strain were centrifuged at 8,000 × *g* for 10 min, and then the pellets were resuspended twice in sterilized washing solution (5 mmol/L caffeine, 0.1 mol/L MgSO_4_·7H_2_O, 0.03% Tween 80) for an absorbance of 0.4 at a 550 nm wavelength. A 1.5 mL suspension of *B*. *velezensis* M1891 and UV4-II was loaded on watch glasses (9 cm in diameter), which were placed 25 cm from a UV lamps in a UV box with a door [50 cm (W) × 85 cm (H) × 42 cm (D)]. Then, the *B*. *velezensis* M1891 and UV4-II suspension were UV irradiated with 2.8 mW/cm^2^, as measured with a UVX radiometer (UVP Co., Upland, U.S.A.), for a 0.1% survival rate. To select the UV mutants with high iturin yields, 100 μL of each diluted UV-irradiated or mock-treated cell suspensions was spread on an LB agar plate and incubated at 30°C for 1–2 days. A mycelial suspension of *F*. *oxysporum*, which was cultivated in potato dextrose broth at 25°C for 3 days, homogenized by a waring blender, and adjusted to an absorbance of 1.5 at a 550 nm wavelength, was added as a soft overlay of 0.8% PDA onto colonies of *B*. *velezensis* grown on LB agar plates, and then the plates were incubated at 25°C for 2 days. Colonies showing a larger zone of inhibition against *F*. *oxysporum* than that of the mother strain on plates were selected. The selected colonies were cultivated in complex medium at 30°C for 48–72 h, and the culture broths of each strain were then centrifuged at 15,000 × *g* for 10 min. Sterile stainless steel cylinders (8 mm outer diameter × 10 mm long, Fisher) were placed on the surfaces of the agar plates seeded with mycelial suspensions of *F*. *oxysporum*, and supernatants from the wild-type and mutant strain cultures were loaded into the sterile cylinders, and then the plates were incubated at 25°C for 2 days. The diameters of the inhibitory zones on the plates were measured and recorded in millimeters. A mutant strain from *B*. *velezensis* UV4-II showing high yields of iturin was selected for further analysis.

### *In vitro* antifungal activity against *F*. *oxysporum*

To examine the antifungal activity of the wild-type, UV mutant, and other *Bacillus* strains, which were isolated from commercially available products, these strains are inoculated in LB broth and cultured at 30°C for 16 h. Aliquots of the culture broths of each strain were inoculated in complex medium and incubated at 30°C for 72 h. The culture broths of the strains were then centrifuged at 15,000 × *g* for 10 min. The supernatants of the strains were loaded onto paper disks or sterile stainless steel cylinder placed on the surface of the agar plates seeded with mycelial suspensions of *F*. *oxysporum* and incubated at 25°C for 2 days. The diameter of the inhibitory zone of each strain was measured in millimeters.

### Inhibition of spore germination of *Botrytis cinerea*

To examine the suppressive effects of the selected mutant and wild-type *B*. *velezensis* strains on the spore germination of *Botrytis cinerea*, broths from 3-day cultures were centrifuged at 8,000 × *g* for 10 min, and the supernatants were adjusted to pH 3.0 by adding 5 mol/L HCl. The acid precipitates were stored at 4°C overnight and then centrifuged at 8,000 × *g* for 10 min, dissolved in 1 mol/L Tris-HCl (pH 7.4), and fractionated three times with butanol. The butanol layers were evaporated *in vacuo*, dissolved in methanol, and then filtered through a 0.45-μM filter. The spores of *Botrytis cinerea* were cultivated at 25°C for 3 days and then collected by filtration of the culture broths through cheesecloth. The concentration of *B*. *cinerea* spores was adjusted to 10^6^ spores/mL, and aliquots of the butanol extracts were added to each hole of a slide glass at 125–500 μg/mL. The slide glasses with each strain were incubated at 25°C for 6 h, and spore germination rates were observed under a microscope.

### Analysis of the expression levels of iturin and surfactin biosynthetic genes by real-time PCR

The expression levels of genes associated with iturin and surfactin biosynthesis in the mutant strain yielding high levels of iturin were compared with those of the wild-type strain by real-time PCR. Aliquots of the broths of each strain cultured overnight were inoculated into complex medium for iturin production and cultivated at 30°C for 3 days. Then, total RNA was isolated by adding Trizol (Life Technologies). Five units of DNaseI (Fermentas) was added to each RNA extract, and the mixture was incubated at 37°C for 30 min to remove genomic DNA. cDNAs were synthesized with 1 μg of DNA-free genomic RNA using RevertAid reverse transcriptase (Life Technologies, USA). One microliter of first-strand cDNA was added to the real-time PCR mixture containing 2× Real-Time PCR Smart Mix (SolGent). PCR amplification of iturin biosynthetic genes was carried out using the following specific primers: ituD-F4 (5´- GTT TGA AGA AGC GAG CGA TG-3´) and ituD-R4 (5´-GGC TTC ACC CCT ATT TCC TG-3´), ituA-F2 (5´-GAC TTC TGA CGG CTG GGT AA-3´) and ituA-R2 (5´-CGT TCA ATA TCG TGC GGG TAG-3´), ituB-F3 (5´-AGT CGC CAT TCT CGC TGA-3´) and ituB-R3 (5´-CAA GGA GAA CGT CTG CAT CC-3´), ituC-F3 (5´-GAT CTT CGT TCA GAC CAG CTC-3´) and ituC-R3 (5´-GCA TTG TAG TTC AGC CTC AGC-3´). In addition, the following PCR primers for surfactin and *gyrB* genes were used: srfA-F1 (5´-CGG CGG TAT GAG TCG ATG-3´) and srfA-R1(5´-GCT TGC TTG CCT CGT CAC-3´), srfB-F1 (5´-AGA CCG AGG AGG AAC AGC AG-3´) and srfB-R1 (5´-CAG CAG GGA CGT TGT AGC TC-3´), srfC-F1 (5´-GAC CGG TCA AGC TGT TCG-3´) and srfC-R1 (5´-CTT CAT CAG CGC CTG GAC-3´), srfD-F1(5´-CCC GCT CCA CAC CTA TCT TC-3´) and srfD-R1(5´-CTG TGG CCG AAC AGG ACA-3´), gyrB-F1 (5′-GGC TCT CGG GAC AGG AAT-3′) and gyrB-R1 (5′-GGC GGC TGA GCA ATG TAG-3′). Real-time PCR was performed on a Bio-Rad CFX Connect Real-Time PCR System as follows: one initial cycle of denaturation at 95°C for 10 min; 40 cycles at 95°C for 30 s, 58°C for 30 s, followed by 72°C for 15 s. The *gyrB* gene, which encodes the subunit B protein of DNA gyrase, was used as the control. To analyze the relative changes in gene expression, the 2^–(ΔΔCt)^ method was used [33]. The relative expression levels of iturin and surfactin genes were derived by subtraction of cycle threshold (Ct) value of the *gyrB* gene used as an internal control gene from the Ct values of each iturin and surfactin biosynthesis gene.

### Analysis of secondary metabolites by HPLC

To compare iturin, surfactin, and fengycin production by the wild-type and mutant strains, these strains were grown in complex media at 30°C for 3 days. The culture broths were centrifuged at 8,000 × *g* for 10 min, and the supernatants were adjusted to pH 3.0 to precipitate cyclic lipopeptides at 4°C overnight. The precipitates were centrifuged, dissolved in 1 mol/L Tris-HCl buffer (pH 7.4), and extracted three times with butanol. The butanol layers were evaporated *in vacuo*, dissolved in methanol, and then filtered through a 0.45-μM filter. The secondary metabolites obtained from the culture broths of the wild-type and mutant *B*. *velezensis* strains were analyzed by HPLC (Agilent 1100) with a C_18_ column (YMC-pack Pro, 4.6 × 250 mm, 5 μm; YMC). The peaks at 210 nm were detected by UV detector. The column was eluted with a gradient of CH_3_CN (A)/0.05% trifluoroacetic acid (TFA) in water (B) at a flow rate of 1-mL/min as follows: 20–60% of A/80-40% of B (v/v) for 50 min, 60–80% of A/40-20% of B (v/v) for 5 min, 80–100% of A/20-0% of B (v/v) for 30 min, 100% of A/0% of B (v/v) for 3 min, and 20% of A/80% of B (v/v) for 2 min. Iturin and surfactin reference standards (Sigma) were used for comparison with products from the wild-type and mutant strains.

### Evaluation of *in vivo* antifungal activity in the greenhouse

The *in vivo* antifungal activity of the mutant strain against fusarium wilt (*Fusarium oxysporum*) of tomato (*Lycopersicon esculentum* Mill, cv. Hoyong) plants was examined using soil naturally infested with *F*. *oxysporum* in a commercial greenhouse (0.9 m × 5 m each, 17 plants/plot × three plots) near Ganghwa-gun, Incheon Metropolitan city, Korea. Experiments of the antifungal activity of the mutant against sclerotinia rot (*Sclerotinia sclerotiorum*) of lettuce (*Lactuca sativa* L, cv. Jeokchukmyun) were carried out using a naturally infested soil containing *S*. *sclerotiorum* in a commercial greenhouse (1.5 m × 5 m each, 170 plants/plot × three plots) near Yangpyung-gun, Gyunggi-do prefecture, Korea. Effective fungicides against *F*. *oxysporum* and *S*. *sclerotiorum* were not used during the experimental periods, and each experiment was performed under natural inoculum pressure. The experiments used a randomized complete block design with three rows, containing six treatments per row. Each treatment plot was 5 m long, but the data were collected in the middle of 4 m, leaving 0.5 m on either side as an interval between treatment plot. For the fusarium wilt and sclerotinia rot bioassay, both plants were inoculated by leaf spraying and soil drench using the culture broth of mutant strain that was diluted 500–1,000-fold from culture broth (1 × 10^9^ CFU/mL) cultivated at 30°C for 32 h in a 5,000 liter fermenter (working volume 3,500 liter, 0.5 vvm, 50 rpm, pH 7.0 ± 0.5), according to farmers' conventional practice, approximately every week. Spraying was performed three times with a hand-held sprayer until run-off. Disease severity was examined at 7-day intervals after spraying just prior to harvest and the percentage of disease incidence was evaluated as the mean of triplicate independent experiments. The antifungal activity of the mutant strain was also examined at 7-day intervals just prior to harvest in three independent experiments. The average value of three estimates for each treatment was converted into a percentage of fungal control as previously described [34]: % control = 100 × [(A—B)/A], where A = area of infection (%) on leaves or sheaths sprayed with Tween 20 solution alone and B = area of infection (%) on treated leaves or sheaths. The antifungal activities of the mutant strain against phytopathogenic fungi were compared with those of the positive control. Disease-control values were expressed as percentage control (± standard deviation) compared to the control.

### Statistical analysis

Statistical analysis was conducted using the MYSTAT12 procedure (Systat software Inc, Chicago, IL, USA), and the means were determined by the least significant difference test at the *P* = 0.05 levels.

## Results

### Taxonomic position of the wild-type *Bacillus* strain

To further analyze the wild-type *Bacillus subtilis* subsp. *krictiensis* strain identified by phenotypic characteristics in a previous study [30], a phylogenetic analysis of 16S rRNA gene sequences of the wild-type strain against the indicated known sequences was performed. The 16S rRNA gene sequences of the wild-type strain showed a 99.79–100% similarity with many *B*. *amyloliquefaciens* strains (S1 Table). In particular, the wild-type strain shared a 99.93% similarity with *B*. *amyloliquefaciens* subsp. *plantarum* CAU B946 using NCBI BLAST. Phylogenetic tree construction was based on the 16S rRNA gene sequences and demonstrated that this strain was closely affiliated with *B*. *amyloliquefaciens* subsp. *plantarum* CAU B946 (S1 Fig). Finally, *B*. *amyloliquefaciens* subsp. *plantarum* CAU B946 (later *B*. *methylotrophicus* CAU B946) is reported to be a heterotypic synonym of *B*. *velezensis*, based on phylogenomics [35–37]. Taken together, the wild-type strain was reassigned as *B*. *velezensis* KRF-001.

### UV irradiation and selection of a high iturin yielding *B*. *velezensis* mutant

To obtain a UV mutant with increased iturin production, the UV mutants M1891 and UV4-II strains were reirradiated by UV. The optimal time for UV treatment resulting in a 0.1% survival rate for M1891 and UV4-II strains were 25 min and 30 min, respectively (S2 Fig).

After UV treatment, suspensions of *B*. *velezensis* M1891 and UV4-II were spread on LB agar plate. The colonies of mutants grown on agar plates were prepared in duplicate using the replica plating method [38], and one of the two plates was used for the bioassay. Soft agar containing the mycelial suspension of *F*. *oxysporum*, which showed a zone of inhibition in respond to iturin, but not to surfactin [31], was overlaid on the colonies of *B*. *velezensis* grown on LB agar plates. Finally, one colony derived from the *B*. *velezensis* UV4-II strain was selected from the colonies of 30,000 mutants that showed higher antifungal activity than that of the mother strain. This colony was designated a high iturin yielding mutant *B*. *velezensis* BSM54, and was used for further studies.

### Comparison of antifungal activities of commercial products and *B*. *velezensis* mutants

To confirm the antifungal activity of mutant *B*. *velezensis* BSM54 against *F*. *oxysporum* as described previously [31], the potency of this strain was compared with the activities of *Bacillus* strains isolated from various commercially available products, including Serenade^®^ and Kodiak^®^ from domestic and foreign countries. The strains isolated from commercial products and mutant strain BSM54 were cultivated in complex medium at 30°C for 3 days to produce cyclic lipopeptides, and the supernatants of each strain were used in bioassays against *F*. *oxysporum*. The strains from commercial products showed inhibition zones of 13.2 to 14.3 mm against *F*. *oxysporum*, whereas the three UV mutants from the wild-type strain showed zones of 14.4 to 16.5 mm. Among these mutants, *B*. *velezensis* strain BSM54 showed the most potent antifungal activity against *F*. *oxysporum* (S3 Fig).

### Suppressive effects of mutant *B*. *velezensis* BSM54 on spore germination of *B*. *cinerea*

**Fig 1:**
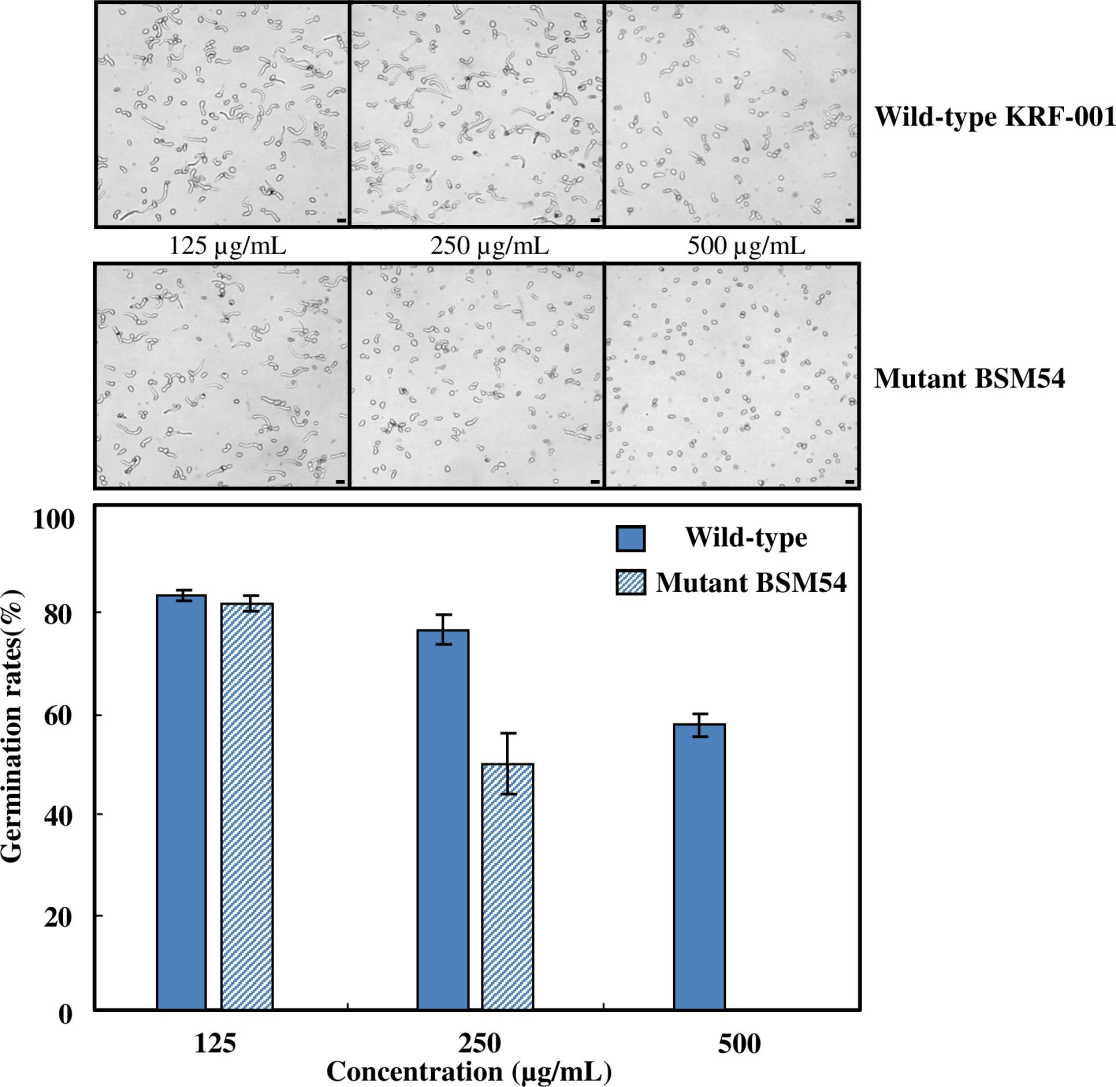
Comparison of the spore germination rates of *Botrytis cinerea* treated with butanol extracts prepared from the culture broths of wild-type *Bacillus velezensis* and mutant strain BSM 54. Bars indicate 20 μm.

To investigate the suppressive effects of a high iturin yielding mutant BSM54 and wild-type strains on spore germination of *B*. *cinerea*, the causative agent of cucumber gray mold, the butanol fractions obtained from culture broths of each strain were used in a spore germination assay. Effects of the wild-type and mutant BSM54 strains producing iturin and surfactin were not significantly different when butanol fractions were used in treatments at the same concentration of 125 μg/mL. However, mutant strain BSM54 inhibited 51.32% of spore germination at a concentration of 250 μg/mL, showing 2-fold stronger activity than that of the wild-type strain. Moreover, the spore suppressive rate of the wild-type strain was 43.54% at a concentration of 500 μg/mL, whereas spore germination failed to occur at the same concentration of the mutant strain. These results indicate that mutant strain BSM54 suppresses spore germination of *B*. *cinerea* more potently than does the wild-type strain (Fig 1).

### Comparative analyses of the expression levels of iturin and surfactin biosynthesis genes in mutant strain BSM54 by real-time PCR

**Fig 2:**
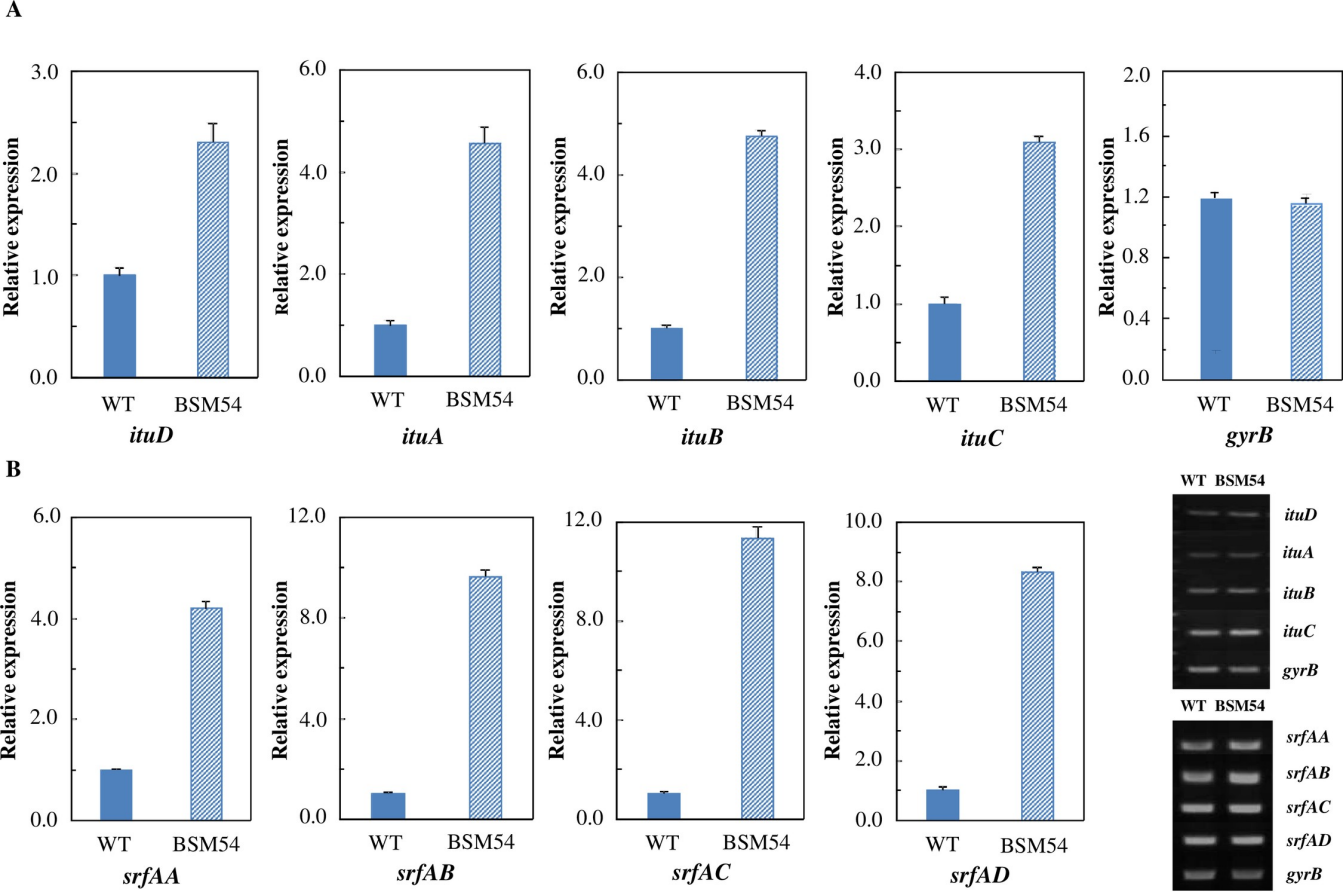
Expression levels of iturin (A) and surfactin (B) biosynthesis genes determined by real-time PCR of the genomic DNA of wild-type *Bacillus velezensis* and mutant strain BSM54 cultivated for 72 h at 30°C. The data are expressed as mean ± SD for three separate experiments. The *gyrB* gene, encoding for the B subunit of the DNA gyrase, was used as the control.

To investigate whether the expression levels of iturin and surfactin biosynthesis genes had changed, the expression levels of *ituA* to *ituD* and *srfAA* to *srfAD* associated with iturin and surfactin biosynthesis, respectively, were analyzed in the BSM54 mutant and wild-type strains by real-time PCR. Isolation of total RNAs from mutant BSM54 and wild-type strains and real-time PCRs of their cDNA were performed according to the methods described in Materials and Methods. The expression levels of genes *ituA*-*D*, responsible for iturin production, in BSM54 mutant strain were significantly increased above that of the wild-type strain after 72 h, when the maximum antifungal activity against *F*. *oxysporum* was observed in the wild-type strain. Specifically, remarkable differences were observed in the expression levels of the *ituA* to *ituC* genes in the BSM54 mutant and wild-type strains. The expression levels of these genes were about 3.1 to 4.8-fold higher than those of the wild-type strain, whereas the level of *ituD* gene was 2.3-fold higher. In addition, the expression levels of genes *srfAA* to *srfAD* for surfactin in mutant strain BSM54 were also significantly increased above that of the wild-type strain. Among these genes, the expression levels of the *srfAA* to *srfAD* genes in the mutant strain were approximately 4.2 to 11.3-fold higher than those of wild-type strain (Fig 2). Based on these results, the increased antifungal activity of mutant strain BSM54 against *F*. *oxysporum* appears to be due to the increased expression levels of iturin biosynthesis genes, because surfactin did not show antifungal activity against *F*. *oxysporum* in the bioassay [31].

### Comparative analysis of iturin, surfactin, and fengycin metabolites produced by the wild-type *B*. *velezensis* and mutant BSM54 strains

**Fig 3:**
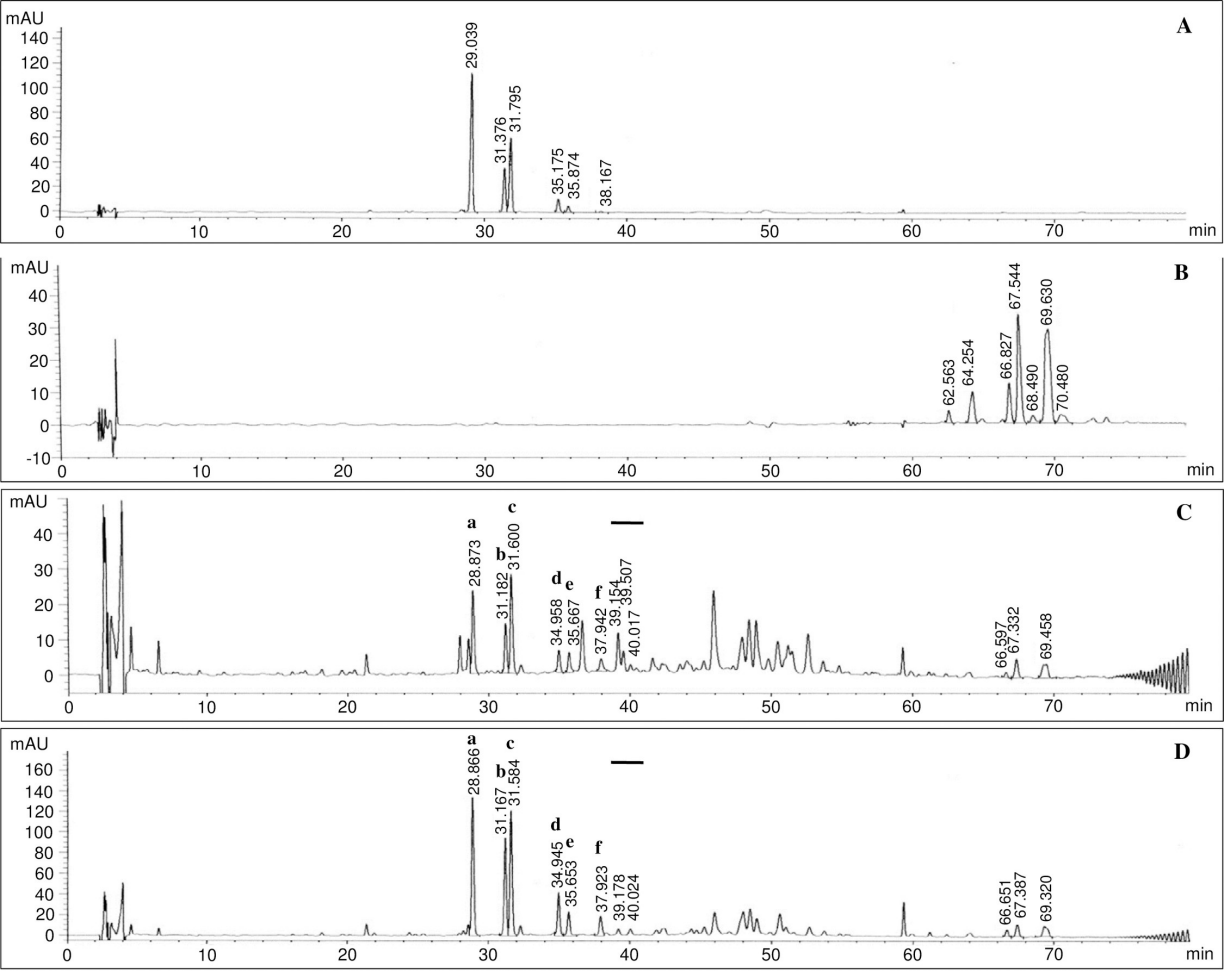
Qualitative HPLC analyses of the iturin, surfactin, and fengycin compounds produced by the wild-type and mutant BSM54 strains. A: Authentic iturin A (500 μg/mL), B: Authentic surfactin (500 μg/mL), C: Wild-type *Bacillus velezensis* KRF-001, D: Ultraviolet mutant *Bacillus velezensis* strain BSM54. *a-f in the chromatograms of Fig 3C and Fig 3D indicate iturins A to F. **Thick solid bars in the chromatograms of Fig 3C and Fig 3D represent the putative fengycin peaks.

The butanol fractions evaporated *in vacuo* from butanol extracts of the wild-type and mutant BSM54 strains were analyzed by HPLC and compared with the HPLC profiles of reference iturin A (Fig 3A) and surfactin (Fig 3B), which were used as controls. Six peaks of iturin compounds were detected in wild-type *B*. *velezensis* (Fig 3C) and its mutant BSM54 strains (Fig 3D), and the patterns of these peaks were very similar to those of the commercially available iturin A. The patterns of these peaks were the same as those of iturins A to F, which were previously isolated and identified in our laboratory [29, 31]. Moreover, small amounts of three surfactin peaks with retention times of 66, 67, and 69 min detected in the wild-type strain were observed in mutant strain BSM54, and iturin and surfactin production by this strain was significantly increased over that of the wild-type strain when the same amounts of butanol extracts were injected in the HPLC analysis (Fig 3D).

In contrast, three peaks of putative fengycin analogues, which were recently detected in the wild-type strain KRF-001 (thick solid bar, Fig 3C), were observed. Only two peaks of putative fengycin analogues in the mutant strain BSM54 (thick solid bar, Fig 3D) were observed. Moreover, small amounts of fengycin analogues were detected in the mutant BSM 54. The mass spectra of the three peaks of putative fengycin analogues detected by HPLC showed quasi-molecular ion peaks [M+H]^+^ at 1,463.7, 1,477.7, and 1,505.7 m/z (see S4–S7 Figs in the supporting information), corresponding to molecular weights of 1462, 1476, and 1504, respectively. The molecular weights of these peaks corresponded to previously reported molecular masses of fengycin A (1,462 and 1,476) and B (1,504) [17, 39, 40]. However, the amount of fengycin obtained from the mutant strain BSM54 was significantly lesser than that of iturin from the same strain and less surfactin was produced when the same amounts of butanol extracts were injected into the HPLC instrument (Fig 3D). This suggests that the increased antifungal activity of the mutant BSM54 against *F*. *oxysporum* (S3 Fig) might be due to the increase in iturin and surfactin production, even though small amounts of fengycin were produced. In addition, iturin and surfactin are lipopeptides that are known to exert synergistic effects [12]. Based on these results, the quantitative analyses of iturin and surfactin analogues produced by mutant BSM54 were further performed.

**Fig 4:**
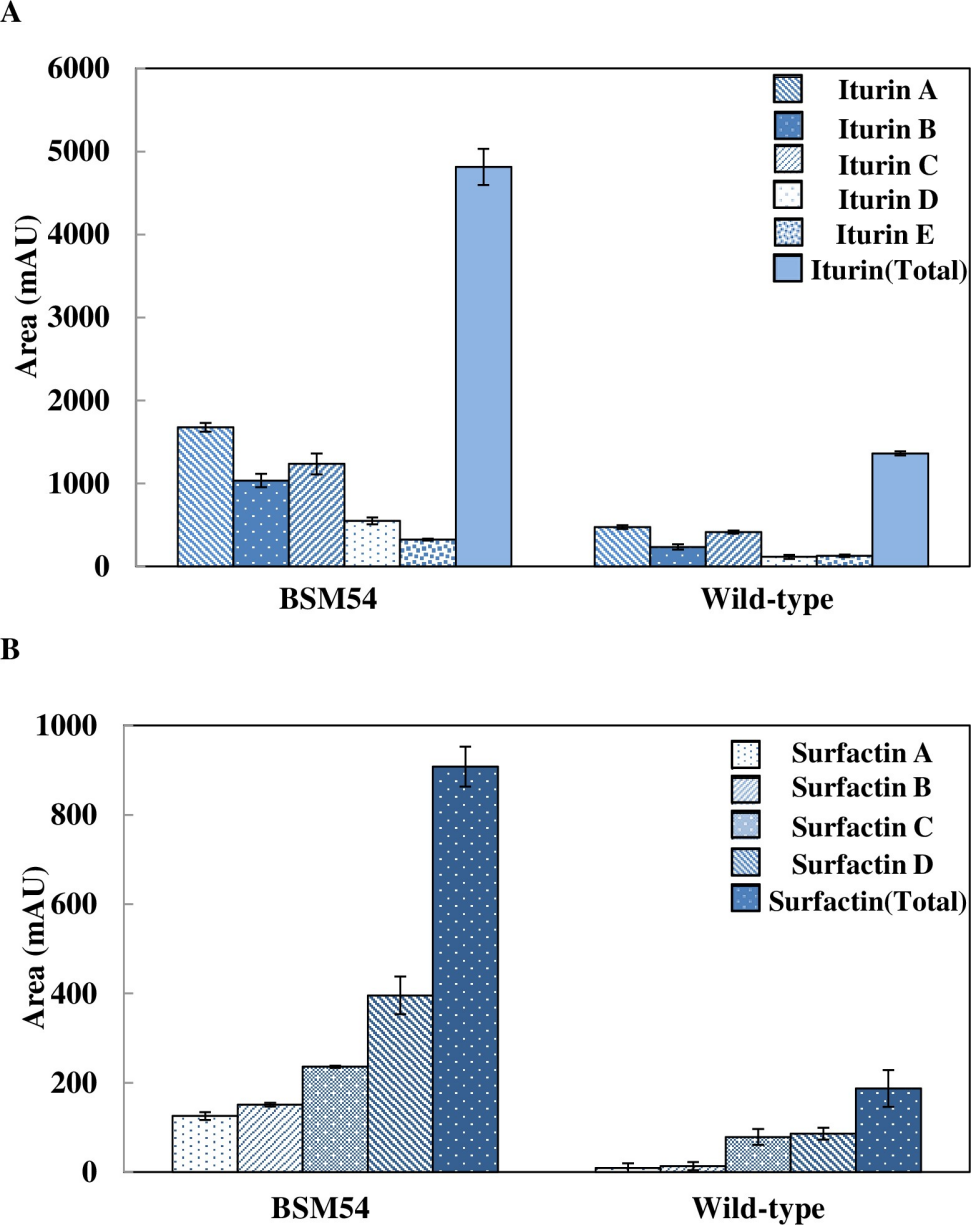
Comparison of HPLC peak areas of iturin (A) and surfactin (B) produced from wild-type *Bacillus velezensis* KRF-001 and its ultraviolet mutant strains. The data are expressed as mean ± SD for independent experiments performed in quadruplicate.

To determine the amount of iturin and surfactin production by mutant strain BSM54, the HPLC peak areas of iturin and surfactin from this strain were compared with those of the wild-type strain. Iturin and surfactin production of mutant strain BSM54 was approximately 3.5 and 5.3-fold higher, respectively, than that of the wild-type strain (Fig 4). Specifically, iturin A production by mutant strain BSM54 was 3.5-fold higher than that of the wild-type strain, although production of iturins B to E was also increased over that of the wild-type strain. Due to the small areas of iturin F peaks in these strains, they were excluded in the comparative analysis. Iturins B and C have been reported to show no antifungal activity [41, 42], whereas iturins A, D, and E show strong antifungal activity [30]. Thus, the enhanced antifungal activity of mutant strain BSM54 against *F*. *oxysporum* was due to increased production of iturins A, D, and E, because surfactin showed no antifungal activity against *F*. *oxysporum* [31].

### Comparative *in vivo* antifungal activity of wild-type *B*. *velezensis* and mutant BSM54 strains

*In vivo* protective activities against fusarium wilt of tomato and sclerotinia rot of lettuce plants were evaluated under commercial greenhouse conditions. The diseases-control efficacy of the *B*. *velezensis* mutant strain against fusarium wilt and sclerotinia rot was 75.4% and 71.1% at 2.0 × 10^6^ CFU/mL, respectively, whereas the wild-type strain showed 62.8% and 63.2% at the same concentration. In addition, the diseases-control values of the mutant against both plant diseases were 60.4% and 62.0% at 1.0 × 10^6^ CFU/mL, respectively, while the wild-type exhibited 56.2% and 54.2% at the same concentration (Table 1). The diseases-control activity of the mutant strain was stronger than that of the wild-type strain and commercial product Cillus^®^ available in Korea. No phytotoxicity during the experiment periods was observed and the average infected rates of fusarium wilt in tomato and sclerotinia rot in lettuce in the commercial greenhouse were observed to be 31.2–42.9% disease incidence, respectively.

**Table 1:** *In vivo* disease-control activities of *B*. *velezensis* mutant BSM54 strain against fusarium wilt of tomato and sclerotinia rot of lettuce.

Treatment^#a^		Control value (%)^#b^	
		*Sclerotinia sclerotiorum*	*Fusarium oxysporum*
Mutant	1,000×	62.0± 5.18^cb^	60.4± 1.50^b^
	500×	71.1± 3.36^a^	75.4± 0.92^a^
Wild-type	1,000×	54.2± 2.86^c^	56.2± 1.17^c^
	500×	63.2± 3.04^ba^	62.8± 1.10^b^
Cillus^#c^	500×	59.4± 5.01^cb^	62.4± 0.87^b^
Control		˗^d^	˗^d^

^#a^1000× and 500×: 1,000-fold dilution (1 × 10^6^ CFU/mL) and 500-fold dilution (2 × 10^6^ CFU/mL) of each culture broth (1 × 10^9^ CFU/mL) produced from mutant BSM54 and wild-type strains, respectively.

^#b^The values were expressed as the mean ± SD for three independent experiments (*P* = 0.05).

^#c^Commercially available Cillus^®^ in Korea was used as a positive control against fusarium wilt and sclerotinia rot.

## Discussion

The use of biological control agents to manage plant diseases has been explored as an alternative to the use of synthetic fungicides [43, 44]. Specifically, *Bacillus subtilis* has been extensively studied as a biocontrol agent of phytopathogenic fungi because of its capacity to produce cyclic lipopeptides [8–10]. Previously, we isolated a strain, *B*. *subtilis* subsp. *krictiensis* ATCC55079, that produced potent cyclic lipopeptides, iturins A to F [29, 31], with the molecular weights of 1042, 1056, 1056, 1070, 1070, and 1084 Da, respectively, indicating 14 mass units higher than the values for iturin A, and a small amount of surfactin, for suppression of various phytopathogenic fungi. Recently, small amounts of fengycin A (molecular weights of 1,462 and 1,476) and B (molecular weight of 1,504) have been observed in the wild-type strain (Fig 3C and S4–S7 Figs). In this study, we reassigned the wild-type *B*. *subtilis* subsp. *krictiensis* ATCC55079 identified by phenotypic characteristics in a previous study [30] as *B*. *velezensis* KRF-001 based on 16S rDNA analysis and phylogenomics results, since *B*. *amyloliquefaciens* subsp. *plantarum* CAU B946 (later *B*. *methylotrophicus* CAU B946) is reported to be a heterotypic synonym of *B*. *velezensis* [35–37]. Moreover, we isolated a UV mutant yielding high levels of iturin and examined its antifungal activity in comparison with that of wild-type *B*. *velezensis* for its potential use in commercial agriculture. The potency of the selected UV mutant was increased 2-fold over that of the wild-type strain. This strain showed stronger inhibition of *F*. *oxysporum* in the same complex medium as that of strains isolated from commercially available products, including Serenade^®^ and Kodiak^®^, although these products had different manufacturing dates. Serenade^®^ and Kodiak^®^, respectively, are used to inhibit various plant diseases worldwide and to prevent diseases in cotton in the United States [45, 46]. Results of our *F*. *oxysporum* bioassay agree with the finding that *Fusarium* spp. are more specific for screening of iturin A-producing strains [43]. The butanol fraction of mutant strain BSM54 showed 2-fold stronger and dose-dependent suppression of spore germination of *B*. *cinerea*, relative to that of the wild-type strain. These results suggest that this UV mutant strain yielding high levels of iturin has a potent suppressive effect on certain phytopathogens, and it may be used as a biological control agent.

The expression levels of the genes associated with iturin and surfactin biosynthesis in mutant strain BSM54 were compared with those of the wild-type strain by real-time PCR. The iturin and surfactin biosynthesis genes were found to be expressed at higher levels in the mutant strain. In agreement with this, iturin and surfactin production in mutant strain BSM54, as determined by HPLC analysis, was approximately 3.5 to 5.3-fold higher, respectively, than that of the wild-type. Interestingly, production of iturin A, known to possess strong antifungal activities among iturins A to F, was specifically increased in mutant strain BSM54, in contrast to small amounts of surfactin and fengycin (Fig 3D). The amount of fengycin analogues obtained from the mutant strain BSM54 was significantly less than that of iturin and surfactin produced (Fig 3D). It should be noted that a mixture of surfactin and iturin produced by some *Bacillus* strains has been reported to improve iturin’s antifungal activity, because surfactin can form mixed micelles with iturin [22, 23, 47]. Based on these results, the increased antifungal activity of mutant strain BSM54 against *F*. *oxysporum* appears to result from an increase in the expression of iturin biosynthesis genes or synergic effects between the iturin and surfactin produced. Moreover, the greenhouse experiment using soil naturally infested with sclerotinia rot and fusarium wilt showed that the mutant strain more effectively reduced the incidence of disease than the wild-type and commercially available Cillus^®^ in Korea. These results suggest that the mutant showing high iturin yield is a potential candidate for the development of a biological control agent in agriculture.

Contrastingly, considering the strong antifungal activity of fengycin against various filamentous fungi [48], the enhanced antifungal activity of mutant BSM54 may partially be attributed to small amounts of fengycin other than iturin and surfactin or interaction between iturin and fengycin, even though the production of fengycin was significantly lower than that of iturin.

In order to localize the mutation generated within the iturin biosynthetic genes (37,249 bp), we compared the *ituA* to *ituD* iturin biosynthetic gene sequences of the mutant and wild-type strains and found no difference between them. Thus, the mutations seem to have occurred outside the iturin biosynthetic genes in the mutant strain, which might be responsible for the increase in the iturin expression level and production. Future work should focus on the whole-genome analyses of both the wild-type and mutant strains to identify the mutations affecting iturin production.

Lipopeptides including iturin, surfactin, and fengycin not only inhibit a broad spectrum of phytopathogenic fungi but also have been widely applied in industry and medicine [43]. In our present study, *B*. *velezensis* strain BSM54, which produces high levels of iturin as well as surfactin and fengycin, showed potent antifungal activity, suggesting that this strain can be exploited as a biocontrol agent. This mutant strain may be used in Korea to reduce the amounts of chemical pesticides or manage fungicide resistance in the future. Finally, interactions between iturin and surfactin or between iturin and fengycin in the *B*. *velezensis* mutant strain should be investigated further.

## Supporting information

S1 Table List of *Bacillus* strains containing 16S rRNA gene sequences displaying ≥ 99.79% similarity to the wild-type KRF-001 strain in the NCBI nucleotide sequence database. (DOCX)

S1 Fig Phylogenetic tree based on the partial nucleotide sequences of 16S rDNA. A neighbor-joining phylogenetic tree of wild-type strain KRF-001 was constructed using MEGA 7.0. The percentage numbers at the nodes indicate the levels of bootstrap values based on a neighbor-joining analysis of 1,000 replications. The scale bar indicates 0.0005 nucleotide substitutions per nucleotide position.
(DOCX)

S2 Fig Survival curves of *Bacillus velezensis* M1891 (A) and the UV4-II (B) mutant obtained from UV-irradiated wild-type *Bacillus velezensis* KRF-001.
(DOCX)

S3 Fig Comparison of the antifungal activities against *Fusarium oxysporum* of commercial products and UV mutants producing iturin. S1 (inhibition zone, 13.2 mm) and S2 (13.7 mm): *Bacillus subtilis* QST713 isolated from the commercial product Serenade^®^, which had a different manufacturing date; S3 (14.3 mm): *Bacillus subtilis* QST713 isolated from a commercial product available in Korea; K (13.8 mm): *Bacillus subtilis* isolated from commercially available Kodiak^®^; C (13.6 mm): *Bacillus subtilis* isolated from a commercial product available in Korea; Wild-type (11.2 mm): *Bacillus velezensis*; M1891 (14.5 mm) and UV4-II (14.4 mm): UV mutants of the wild-type strain; BSM 54 (16.5 mm): UV mutant newly obtained from the UV irradiation of mutant UV4-II.
(DOCX)

S4 Fig HPLC spectra and molecular weights of fengycin peaks obtained from wild-type *B. velezensis* KRF-001. (DOCX)

S5 Fig MS spectrum of C16-fengycin obtained from the peak detected at a retention time of 27.79 min on the HPLC chromatogram in S4 Fig. (DOCX)

S6 Fig MS spectrum of C17-fengycin obtained from the peak detected at a retention time of 29.52 min on the HPLC chromatogram in S4 Fig. (DOCX)

S7 Fig MS spectrum of C17-fengycin obtained from the peak detected at a retention time of 30.98 min on the HPLC chromatogram in S4 Fig. (DOCX)

S1 File (DOCX)
