# Supplementary material for: Isolation and characterization of a high iturin yielding Bacillus velezensis UV mutant with improved antifungal activity
Source: PLoS One. 2020 Dec 3;15(12):e0234177. doi: 10.1371/journal.pone.0234177 (PMC7714226; doi:10.1371/journal.pone.0234177)
Supplement: S1 Table — (DOCX) [file pone.0234177.s001.docx]

**S1 Table.** Young Tae Kim et al.

| Strain (Accession number) | Max Score | Total Score | Query Cover  (%) | E- value | Sequence similarity (%) |
| --- | --- | --- | --- | --- | --- |
| *B. amyloliquefaciens* (JQ229807.1) | 2656 | 2656 | 100 | 0.0 | 100.00 |
| *B. amyloliquefaciens* (KX215168.1) | 2647 | 2647 | 99 | 0.0 | 100.00 |
| *B. amyloliquefaciens* (KY328743.1) | 2641 | 2641 | 99 | 0.0 | 99.93 |
| *B. amyloliquefaciens* (KU161297.1) | 2641 | 2641 | 99 | 0.0 | 99.93 |
| *B. amyloliquefaciens* (FJ705346.1) | 2651 | 2651 | 100 | 0.0 | 99.93 |
| *B. amyloliquefaciens* (KF040974.1) | 2641 | 2641 | 99 | 0.0 | 99.93 |
| *B. amyloliquefaciens* subsp. *plantarum* (JN700138.1) | 2643 | 2643 | 99 | 0.0 | 99.93 |
| *B. amyloliquefaciens* subsp. *plantarum* (JF899288.1) | 2636 | 2651 | 100 | 0.0 | 99.93 |
| *B. amyloliquefaciens* subsp*. plantarum* (HQ831421.1) | 2645 | 2645 | 99 | 0.0 | 99.93 |
| *B. amyloliquefaciens* subsp. *plantarum* (JN700138.1) | 2643 | 2643 | 99 | 0.0 | 99.93 |
| *B. amyloliquefaciens* subsp. *plantarum* CAU B946 (HE617159) | 2636 | 26283 | 99 | 0.0 | 99.93 |
| *B. amyloliquefaciens* subsp. *plantarum* FZB 42(CP000560) | 2630 | 24523 | 99 | 0.0 | 99.86 |
| *B. amyloliquefaciens* (KY859771.1) | 2639 | 2639 | 100 | 0.0 | 99.79 |
| *B. methylotrophicus* (HQ662592.1) | 2649 | 2649 | 99 | 0.0 | 100.00 |
| *B. methylotrophicus* (KC790265.1) | 2641 | 2641 | 99 | 0.0 | 99.93 |
| *B. methylotrophicus* (KT902017.1) | 2641 | 2641 | 99 | 0.0 | 99.93 |
| *B. velezensis* (KY962350.1) | 2639 | 2639 | 99 | 0.0 | 99.93 |
| *B. velezensis* (KX129843.1) | 2639 | 2639 | 99 | 0.0 | 99.93 |
| *B. velezensis* (MH000677.1) | 2645 | 2645 | 100 | 0.0 | 99.86 |
