# Supplementary figures and images for: Isolation and characterization of a high iturin yielding Bacillus velezensis UV mutant with improved antifungal activity
Source: PLoS One. 2020 Dec 3;15(12):e0234177. doi: 10.1371/journal.pone.0234177 (PMC7714226; doi:10.1371/journal.pone.0234177)

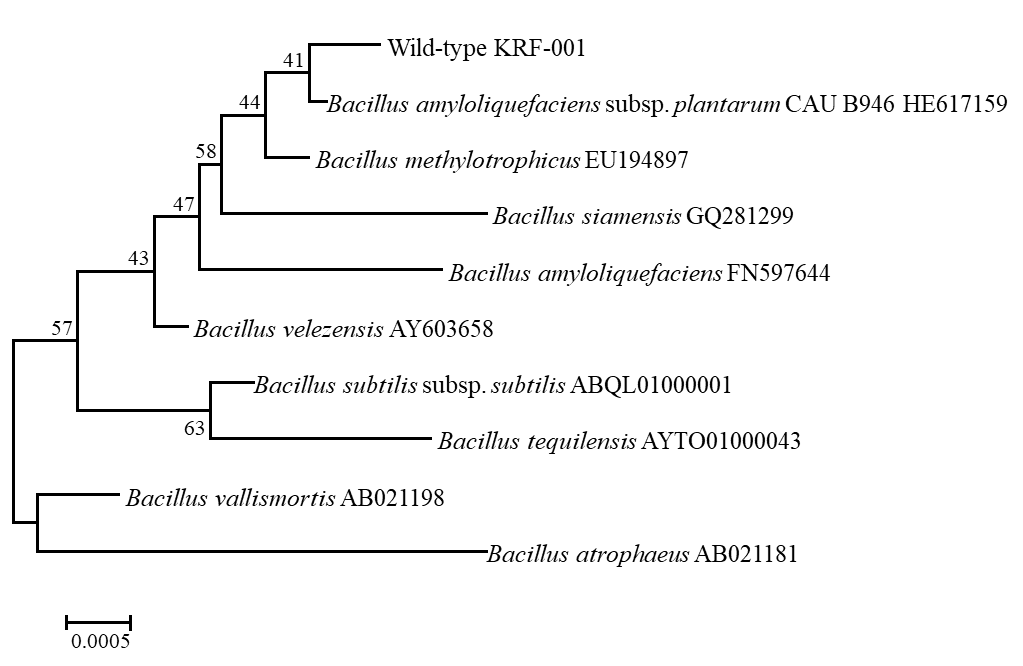


**S1 Fig.** Young Tae Kim et al.

Supplement: S1 Fig — A neighbor-joining phylogenetic tree of wild-type strain KRF-001 was constructed using MEGA 7.0. The percentage numbers at the nodes indicate the levels of bootstrap values based on a neighbor-joining analysis of 1,000 replications. The scale bar indicates 0.0005 nucleotide substitutions per nucleotide position. (DOCX) [file pone.0234177.s002.docx]

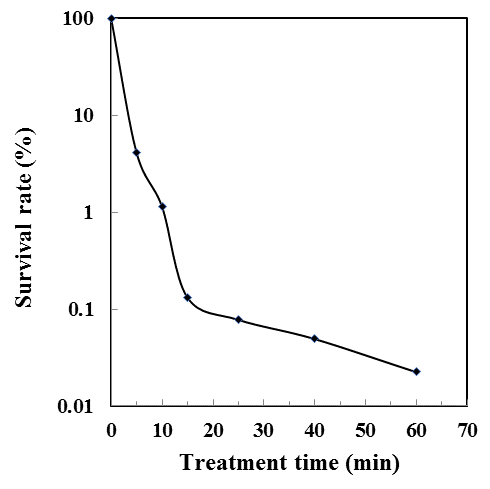

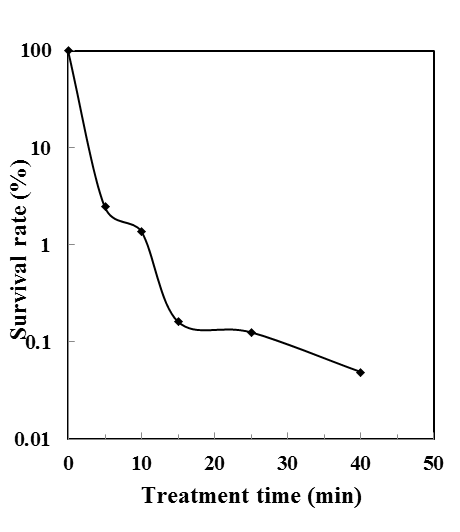


**A**

**B**

**S2 Fig**. Young Tae Kim et al.

Supplement: S2 Fig — Survival curves of Bacillus velezensis M1891 (A) and the UV4-II (B) mutant obtained from UV-irradiated wild-type Bacillus velezensis KRF-001. (DOCX) [file pone.0234177.s003.docx]

**S3 Fig.** Young Tae Kim et al.


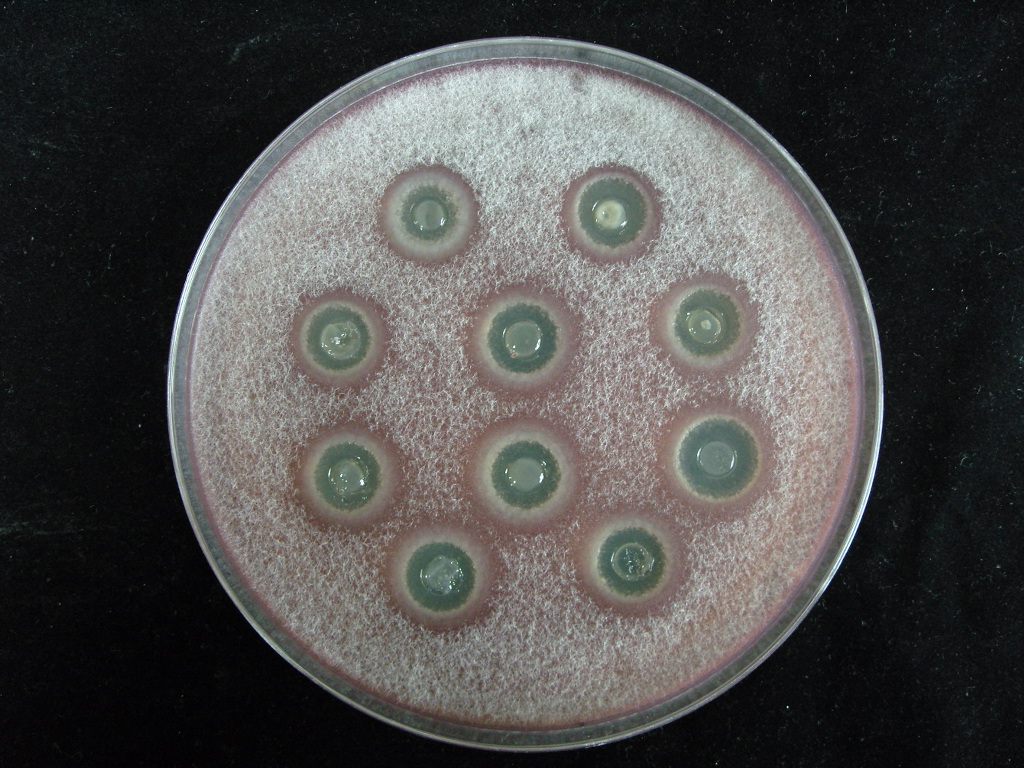


**Wild-type**

**C**

**S1**

**M1891**

**M1891**

**S2**

**K**

**UV4-II**

**BSM 54**

**S3**

Supplement: S3 Fig — S1 (inhibition zone, 13.2 mm) and S2 (13.7 mm): Bacillus subtilis QST713 isolated from the commercial product Serenade®, which had a different manufacturing date; S3 (14.3 mm): Bacillus subtilis QST713 isolated from a commercial product available in Korea; K (13.8 mm): Bacillus subtilis isolated from commercially available Kodiak®; C (13.6 mm): Bacillus subtilis isolated from a commercial product available in Korea; Wild-type (11.2 mm): Bacillus velezensis; M1891 (14.5 mm) and UV4-II (14.4 mm): UV mutants of the wild-type strain; BSM 54 (16.5 mm): UV mutant newly obtained from the UV irradiation of mutant UV4-II. (DOCX) [file pone.0234177.s004.docx]

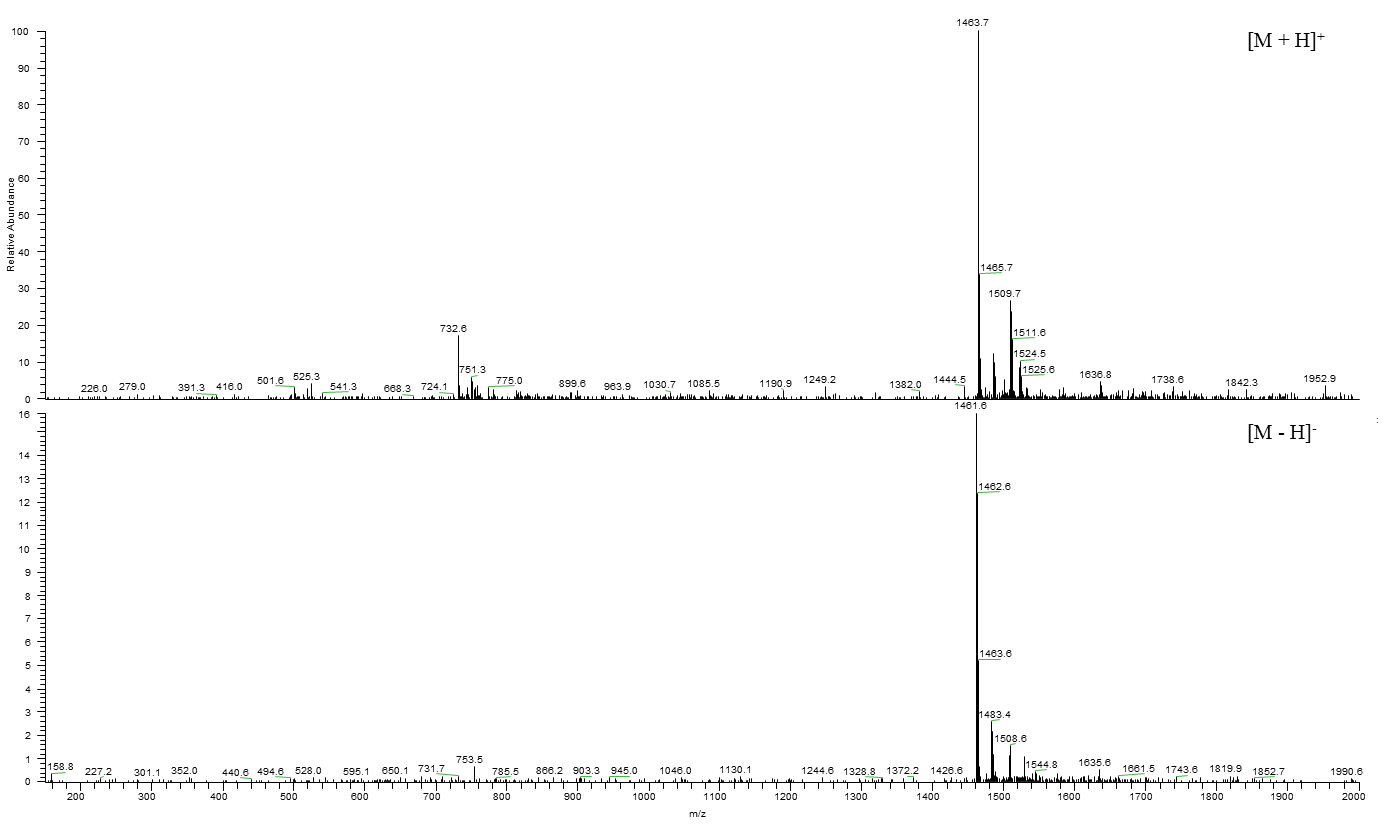


**S5 Fig.** Young Tae Kim et al.

Supplement: S5 Fig — (DOCX) [file pone.0234177.s006.docx]

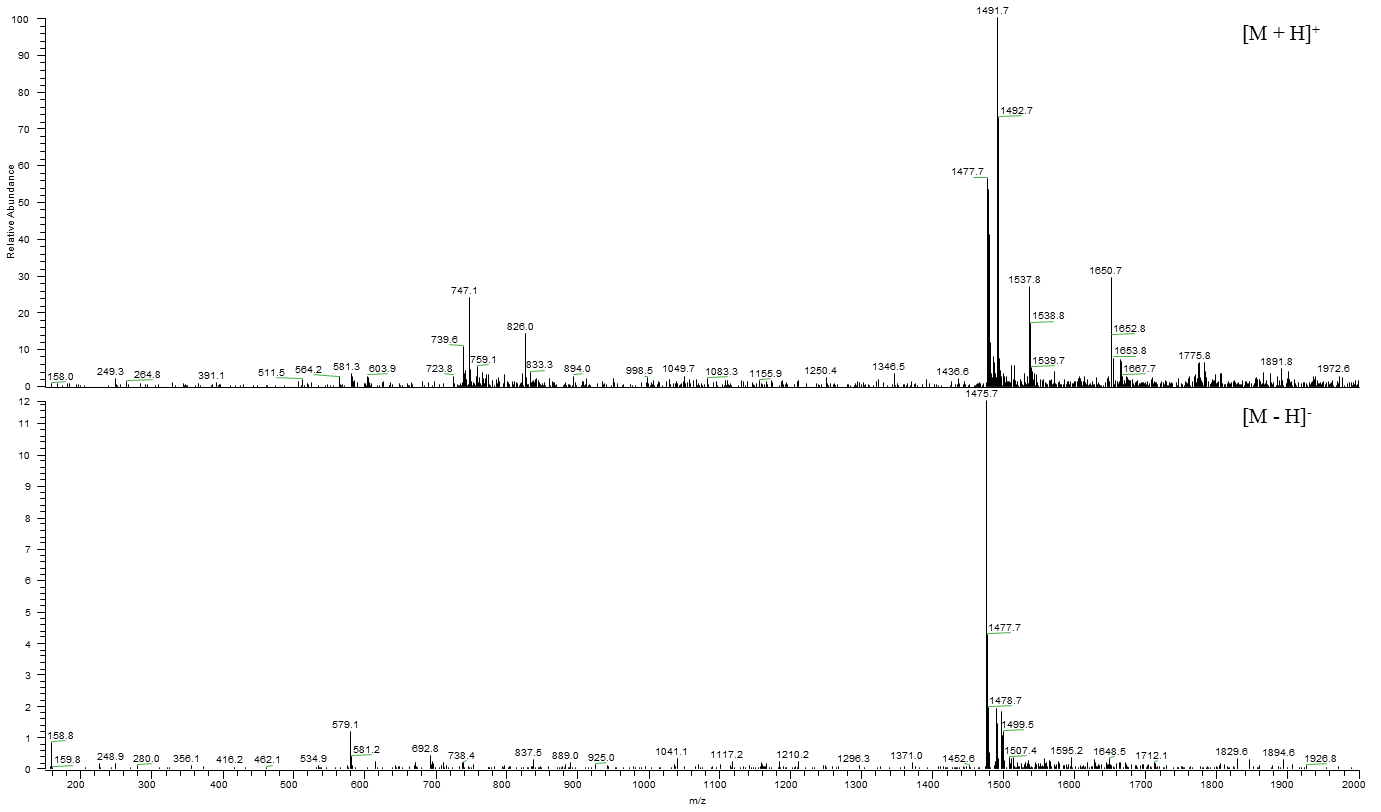


**S6 Fig.** Young Tae Kim et al.

Supplement: S6 Fig — (DOCX) [file pone.0234177.s007.docx]

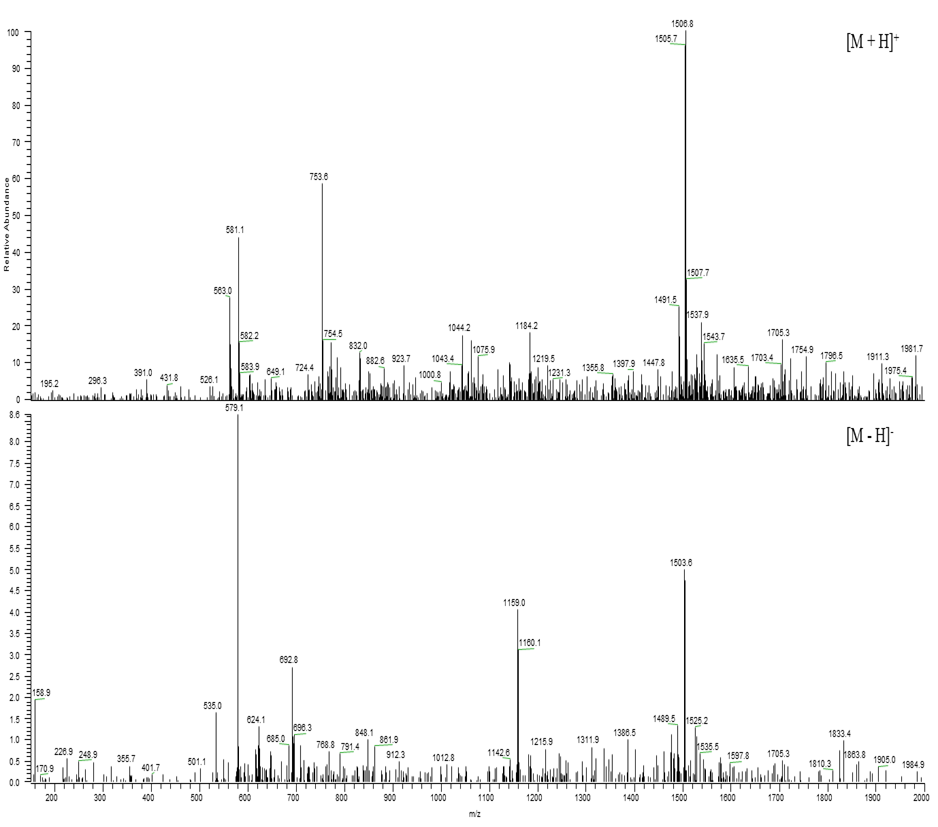


**S7 Fig.** Young Tae Kim et al.

Supplement: S7 Fig — (DOCX) [file pone.0234177.s008.docx]
