# Supplementary material for: Isolation and characterization of a high iturin yielding Bacillus velezensis UV mutant with improved antifungal activity
Source: PLoS One. 2020 Dec 3;15(12):e0234177. doi: 10.1371/journal.pone.0234177 (PMC7714226; doi:10.1371/journal.pone.0234177)
Supplement: S4 Fig — (DOCX) [file pone.0234177.s005.docx]

**S4 Fig**. Young Tae Kim et al.

1462.7

1476.7

1504.8

**Iturin**

**Fengycin**

**Surfactin**
