# Supplementary material for: Isolation and characterization of a high iturin yielding Bacillus velezensis UV mutant with improved antifungal activity
Source: PLoS One. 2020 Dec 3;15(12):e0234177. doi: 10.1371/journal.pone.0234177 (PMC7714226; doi:10.1371/journal.pone.0234177)
Supplement: S1 File — (DOCX) [file pone.0234177.s009.docx]

Supporting information, including list of *Bacillus* strains containing 16S rRNA gene sequences displaying ≥ 99.79% similarity, the phylogenetic tree of the wild-type strain, the survival curves of UV mutants and comparison of the antifungal activities against *F. oxysporum* of several *Bacillus* strains, and the HPLC and ESI-MS spectra used for structural identification of fengycin.
